# ATP in the tumour microenvironment drives expression of nfP2X_7_, a key mediator of cancer cell survival

**DOI:** 10.1038/s41388-018-0426-6

**Published:** 2018-08-07

**Authors:** SM Gilbert, CJ Oliphant, S. Hassan, AL Peille, P. Bronsert, S. Falzoni, F. Di Virgilio, S. McNulty, R. Lara

**Affiliations:** 1 Babraham Research Campus, Biosceptre (UK) Limited, Cambridge, UK; 2 Centre for Cutaneous Research, Blizard Institute, Queen Mary University of London, London, UK; 3 Charles River Discovery Research Services Germany GmbH (formerly named Oncotest GmbH), Freiburg, Germany; 4 Institute for Surgical Pathology, Medical Center—University of Freiburg, Freiburg, Germany; 5 Comprehensive Cancer Center Freiburg, Medical Center—University of Freiburg, Freiburg, Germany; 6 Faculty of Medicine, University of Freiburg, Freiburg, Germany; 7 Section of Pathology, Oncology and Experimental Biology, Department of Morphology, Surgery and Experimental Medicine, University of Ferrara, Ferrara, Italy

**Keywords:** Targeted therapies, Cancer microenvironment

## Abstract

The ATP-gated receptor P2X_7_ is expressed in multiple malignant tumours including neuroblastoma, melanoma, prostate, lung and breast. P2X_7_ has a significant role in mediating diverse cell responses, which upon dysregulation are associated with tumour initiation and development. The rapid, ATP-mediated activation of P2X_7_ induces a fast-inward cation current in cells. However, prolonged ATP-mediated activation of P2X_7_ leads to formation of a pore that increases membrane permeability and eventually causes cell death. This presents a potential paradox, as the tumour microenvironment contains extracellular ATP at levels sufficient to activate the P2X_7_ pore and trigger cell death. However, P2X_7_ expression is associated with enhanced cancer cell survival, proliferation and metastatic potential. At least one distinct conformational form of P2X_7_, termed non-pore functional P2X_7_ (nfP2X_7_), has been described, which is not able to form a functional pore. We demonstrate for the first time in this study that exposure to a high ATP concentration, equivalent to those measured in the tumour microenvironment, drives nfP2X_7_ expression and also that nfP2X_7_ is essential for tumour cell survival. We show that monoclonal antibodies raised against a P2X_7_ amino acid sequence (200–216), whose conformation is distinct from that of wild-type (WT) P2X_7_, bind specifically to nfP2X_7_ expressed on the surface of tumour cells. We also show that nfP2X_7_ is broadly expressed in patient-derived tumour sections from a wide range of cancers. Therefore, antibodies raised against E200 provide tools that can differentiate between forms of the P2X_7_ receptor that have a key role in cancer.

## Introduction

P2X receptors (P2Xs) are ATP-gated cation channels that form homo- and hetero-trimers at the cell membrane [1, 2]. The P2X family comprised of seven members. Although P2X_1_–P2X_6_ are sensitive to ATP concentrations within the nanomolar to low micromolar range, P2X_7_ is less sensitive and requires hundreds of micromolar to millimolar ATP concentrations for activation [2, 3]. P2X_7_ is characterised by a biphasic response [4]. Rapid exposure to ATP trigger opening of a cation-selective channel allowing Na^+^ and Ca^2+^ influx, and K^+^ efflux, whereas prolonged ATP stimulation triggers opening of a non-selective pore permeable to molecules of < 900 Da. Opening of the P2X_7_ pore disrupts intracellular homeostasis, leading to cell death [5–7]. Paradoxically, P2X_7_ activation also drives cytokine release, survival, metabolic adaptations to nutrient deprivation, proliferation, migration and cancer cell invasion [8–11]. Thus, P2X_7_, expressed by cancer cells, can promote a pro-survival and oncogenic outcome rather than facilitating cell death [12–14].

ATP is present at high concentrations (5–10 mM) intracellularly and at very low concentrations in the extracellular compartment of healthy tissues (10–100 nM) [15]. However, in the tumour microenvironment (TME), extracellular ATP (eATP) concentrations can reach hundreds of micromolar [10, 16]. This is due to release of ATP through tumour cell death caused by stresses such as inflammation, hypoxia, mechanical stress and non-targeted therapies [17–19]. In addition, eATP can be increased by cell death-independent mechanisms [15, 18, 19]. Release of ATP is one of the most sensitive danger-associated molecular patterns [15]. The tandem activity of two ectonucleotidases, CD39 and CD73, catalyse eATP hydrolysis to adenosine, thus removing the danger signal. Although high ATP drives inflammation, adenosine is a potent immuno-suppressor [15]. Therefore, the balance between ATP and adenosine orchestrates immunogenicity within the TME.

Tumour cells are exposed to ATP concentrations in the TME sufficient to activate the non-selective pore and precipitate cell death. It was shown previously that in neuroblastoma, P2X_7_ is uncoupled from intracellular cell death-promoting pathways [20]. Indeed, multiple cancer cell types must have developed mechanisms to exploit the trophic advantages mediated by P2X_7_, while minimising the detrimental effects associated with uncontrolled pore opening. Previous reports have identified alternative forms of P2X_7_ termed non-functional P2X_7_ (nfP2X_7_), which do not show large pore function in response to agonist stimulation [17, 21–24]. Polyclonal antibodies raised against the 200–216 amino acid sequence (termed E200) have demonstrated E200 is selectively exposed in nfP2X_7_ but not in wild-type (WT) P2X_7_ [21]. These antibodies have been used to demonstrate strong nfP2X_7_ expression in several cancer types [25–27]. E200 targeting polyclonal antibodies have been developed as therapeutics and show early indications of efficacy against basal cell carcinoma in a phase 1 clinical trial [28] (clinicaltrials.gov NCT02587819).

Here we show that nfP2X_7_ is expressed on multiple cancer cell lines and is necessary for their survival. By mimicking the high ATP concentrations present in the TME, we induce nfP2X_7_ and down-modulate P2X_7_ expression. This provides a mechanism by which tumour cells can maintain the survival advantages, while avoiding cell death induction through opening of the P2X_7_ pore (Fig. 8).

## Results

### P2X_7_ mRNA is expressed in multiple cancer cell lines that do not show pore function

**Fig. 1:**
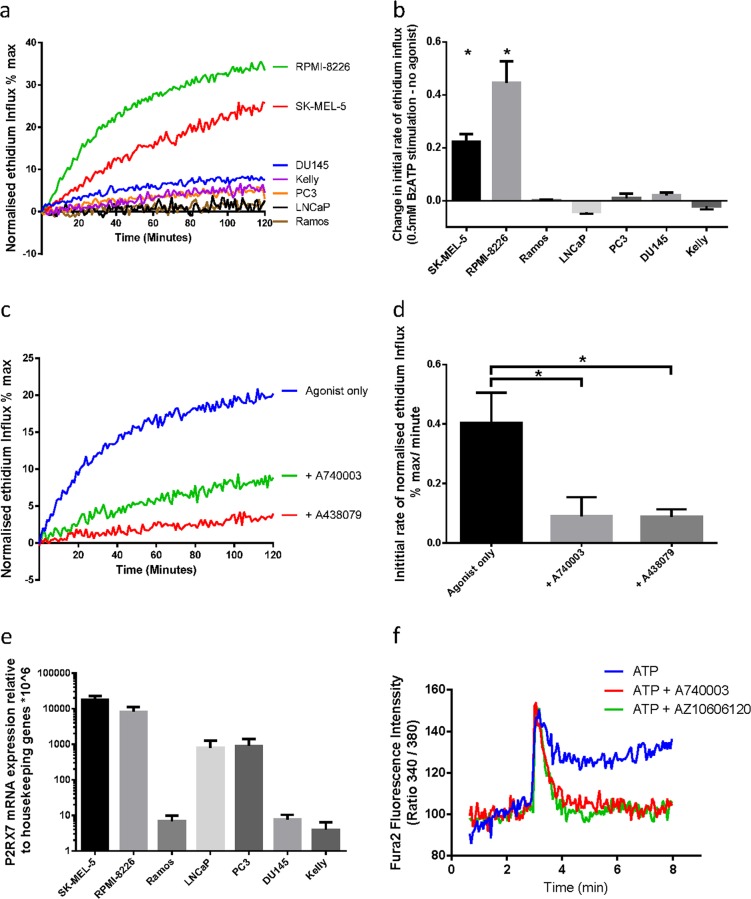
P2X_7_ mRNA is expressed in multiple cancer cell lines which do not show pore function. **a** Normalised ethidium influx in response to 0.5 mM BzATP stimulation in a panel of cancer cell lines. Mean of three independent experiments is shown. **b** Quantification of initial rate (5–40 min) of ethidium influx in 0.5 mM BzATP-treated cells above rate of increase in untreated cells. Mean and SEM from three independent experiments are shown. Two-tailed Student’s *T*-test was used to test whether agonist stimulation caused a significant increase in ethidium influx relative to untreated in each cell line. **c** Normalised ethidium influx in RPMI-8226 cells treated with 0.5 mM BzATP alone or in combination with P2X_7_ inhibitors A740003 or A438079 are shown. **d** Quantification of initial rate (5–30 min) of ethidium influx. Mean and SEM from three independent experiments are shown. One-way ANOVA with Dunnett’s post test was used to test significance. **e** *P2RX7* mRNA quantification by qPCR in a panel of cancer cell lines. Mean and SEM from three independent experiments are shown. **f** Fura-2-loaded PC3 cells were pre-incubated with A740003 or AZ10606120, incubated in a fluorimeter cuvette in standard saline solution and challenged with 3 mM ATP. *P < 0.05

To test the capacity of cancer cells to form the P2X_7_-associated pore, we measured the effect of the ATP analogue, 2′,3′-(4-benzoyl)-benzoyl-ATP (BzATP) on ethidium bromide (EtBr) influx in a panel of cancer cell lines [29]. RPMI-8226 (myeloma) and SK-MEL-5 (melanoma) showed rapid EtBr uptake in response to agonist stimulation (Fig. 1a, b), which was inhibited by pre-incubation with P2X_7_-specific inhibitors A438079 and A740003 (Fig. 1c, d). Other cell lines, including PC3, DU145 and LNCaP (Prostate), Kelly (neuroblastoma) and Ramos (Burkitt’s lymphoma), showed no induction of EtBr influx in response to stimulation with 0.5 mM BzATP (Fig. 1a, b). The absence of EtBr influx was not due to a lack of *P2RX7* transcript in these cell lines, as quantitative PCR (qPCR) analysis demonstrated expression of *P2RX7* mRNA in all cell lines (Fig. 1e). Cell lines with P2X_7_ pore functionality such as SK-MEL-5 and RPMI-8226 showed the highest *P2RX7* mRNA expression levels. PC3 and LNCaP cells, which showed no pore function, also expressed *P2RX7* mRNA. DU145, Kelly and Ramos also showed no pore function, expressing low yet detectable levels of *P2RX7* transcript. We further tested whether ion channel functionality was retained in nfP2X_7_. PC3 cells respond to ATP stimulation with a fast calcium influx, typical of the activation of P2Y receptors, followed by a more sustained calcium influx that was blocked by two specific P2X_7_ inhibitors A740003 and AZ10606120 (Fig. 1f). This suggests that nfP2X_7_ can function as an ion channel. These data show that cancer cell lines with no pore function express *P2RX7* transcript, and that nfP2X_7_ retains ion channel function.

### E200-targeted antibodies specifically bind nfP2X_7_

**Fig. 2:**
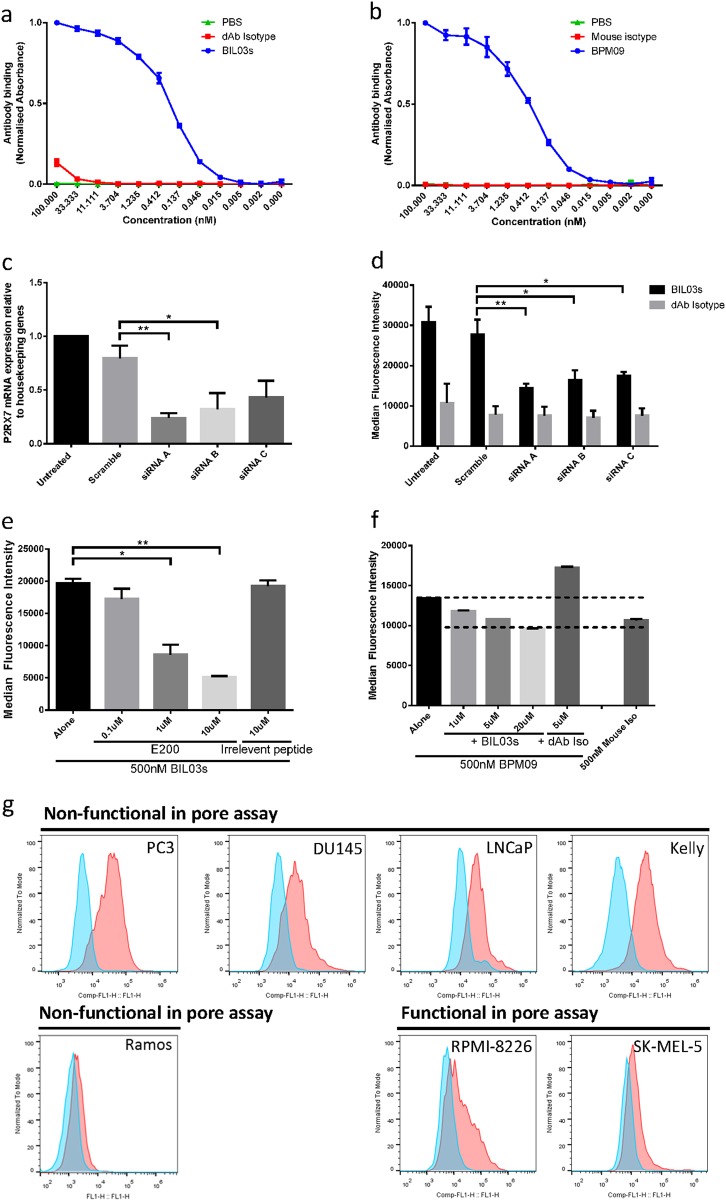
E200-targeted antibodies specifically bind nfP2X_7_. **a**, **b** ELISA assay of BIL03s (**a**) and BPM09 (**b**) binding to E200 peptide compared with PBS and isotype controls. Results were normalised to maximum binding. Mean and SEM from three independent experiments are shown. **c** Quantification of *P2RX7* transcript expression relative to a panel of housekeeping genes in PC3 cells 72 h after transfection with *P2RX7*-targeted siRNA. Mean and SEM from four independent experiments are shown. Each experiment was normalised to untreated control. One-way ANOVA with Dunnett’s post test was used to test significance. **d** BIL03s binding to live PC3 cells 72 h after transfection with *P2RX7*-targeted siRNA was tested by flow cytometry. Mean and SEM from four independent experiments are shown. Two-way ANOVA with Dunnett’s post test was used to test significance. **e** BIL03s binding to live PC3 cells, measured by flow cytometry, can be competed by E200 peptide. Mean and SEM from three independent experiments are shown. One-way ANOVA with Dunnett’s post test was used to test significance. **f** Flow cytometry analysis shows that BPM09 binding to live PC3 cells can be competed with increasing amounts of BIL03s. Representative plot from three experiments are shown. **g** Representative flow cytometry plots from three experiments showing BIL03s binding to live cells in a panel of cancer cell lines. *P < 0.05, **P < 0.01

Two antibodies were raised against the E200 sequence: a fully humanised single-chain domain antibody, designated BIL03s [30], and a mouse hybridoma-derived monoclonal antibody, designated BPM09. Both antibodies were shown by enzyme-linked immunosorbent assay (ELISA) to bind dose dependently to the E200 peptide (Fig. 2a, b). To confirm the specificity of these antibodies for nfP2X_7_ we used *P2RX7*-targeted small interfering RNA (siRNA). Three different siRNA oligonucleotides strongly inhibited *P2RX7* transcript expression and BIL03s binding to PC3 cells (Fig. 2c, d). The same outcomes were observed for BPM09 (data not shown). We then confirmed that BIL03s binding to the E200 sequence on nfP2X_7_ was through its complementarity-determining regions by competing its binding with increasing amounts of E200 peptide, while an irrelevant peptide control had no effect (Fig. 2e). We investigated by flow cytometry the ability of BIL03s to compete for binding of BPM09 to PC3 cells (Fig. 2f). BIL03s reduced BPM09 binding in a dose-dependent manner compared with isotype control. Overall, our data demonstrates that both BIL03s and BPM09 bind selectively to E200 exposed on nfP2X_7_ at the surface of PC3 cancer cells.

To confirm surface expression of nfP2X_7_, we assessed binding of BIL03s to a panel of cell lines by flow cytometry (Fig. 2g). Cell lines demonstrating low EtBr influx (Fig. 1a), such as PC3, DU145, LNCaP and Kelly showed increased BIL03s binding compared with fully functional cell lines, RPMI-8226 and SK-MEL-5 (Fig. 2g). There was low correlation between *P2RX7* mRNA expression and BIL03s binding. However, we did not anticipate a close correlation between BIL03s binding and transcript level, as most of the transcript might be translated into P2X_7_ in some cell types or nfP2X_7_ in other cell types.

### nfP2X_7_ is distinct from functional P2X_7_

**Fig. 3:**
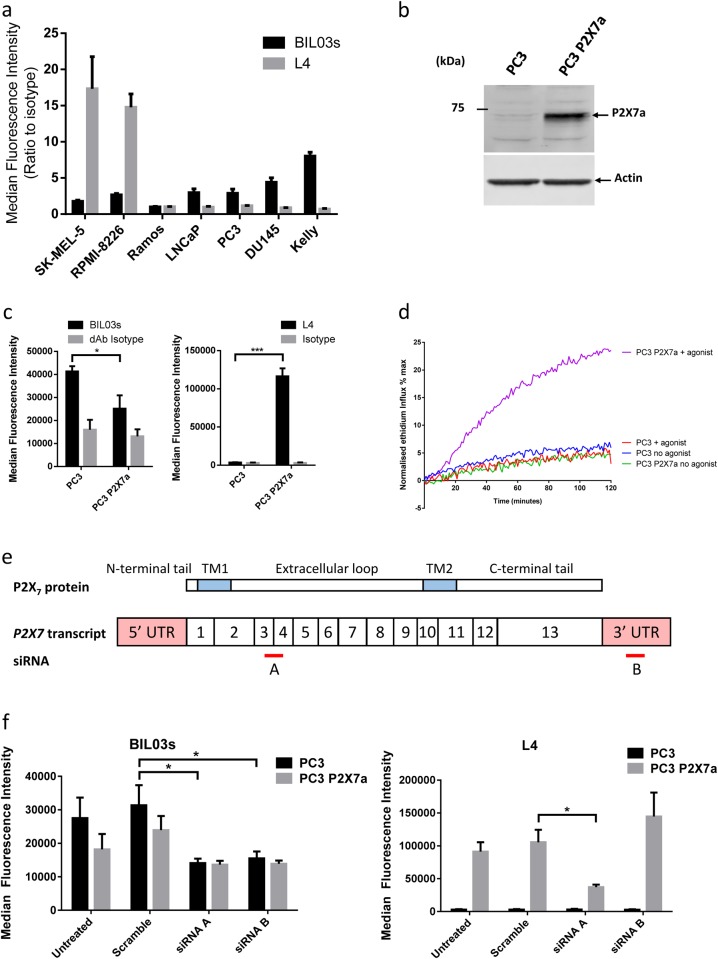
nfP2X_7_ is molecularly distinct from functional P2X_7_. **a** BIL03s and L4 binding relative to isotype control was measured by flow cytometry in a panel of cancer cell lines. Mean and SEM from at least three independent experiments are shown. **b** Western blotting of lysate from PC3 cells or PC3 cells overexpressing P2X_7_a. Representative images from three independent experiments are shown. **c** PC3 cells overexpressing P2X_7_a show increased L4 binding relative to untransfected PC3 but no change in BIL03s binding. Mean and SEM from three independent experiments are shown. Two-way ANOVA with Sidak’s multiple comparison test was used. **d** Ethidium influx into PC3 cells or PC3 cells overexpressing P2X_7_a in response to 0.5 mM BzATP or no agonist stimulation. Mean of three independent experiments shown. **e** Representation of the *P2RX7* transcript and the target sequence location for siRNA. **a**, **b**, **f** The effect of *P2RX7*-targeted siRNA A and B on BIL03s and L4 binding in PC3 cells and PC3 cells overexpressing P2X_7_a was measured by flow cytometry. Mean and SEM from three independent experiments are shown. Two-way ANOVA with Dunnett’s post test was used to test significance. *P < 0.05, ***P < 0.001

To evaluate the relative amount of P2X_7_ and nfP2X_7_ at the cell surface, we investigated binding of BIL03s and the anti-P2X_7_ monoclonal L4 antibody, relative to isotype control in a panel of cancer cell lines (Fig. 3a). L4 was generated by immunisation of mice with a syngeneic cell line overexpressing human P2X_7_ variant a (P2X_7_a) [31]. Ramos cells showed no binding of nfP2X_7_-specific antibodies BIL03s (Fig. 2c), BPM09 (data not shown) or anti-P2X_7_a L4 antibody in agreement with previous reports showing that these cells do not express P2X_7_ [32]. BIL03s bound to all other cancer cell lines tested between two- and eight-fold above isotype control (Fig. 3a), suggesting that nfP2X_7_ is expressed broadly in many cancer cell types as observed previously [25–27]. Comparing L4 and BIL03s binding with EtBr uptake (Figs. 1a, b and 3a) showed that high L4 and low BIL03s binding correlates with the capacity of cells to open the non-selective pore (Fig. 3a). Conversely, cell lines with no functional pore activity showed low L4 and higher BIL03s binding (Fig. 3a). These observations suggest that BIL03s and L4 bind to distinct forms of P2X_7_.

To confirm that BIL03s does not recognise WT P2X_7_, we stably overexpressed P2X_7_a in PC3 cells and sorted the high expressing cells based on L4 binding. P2X_7_a overexpression and L4 binding to the selected population was confirmed by western blotting, flow cytometry and immunofluorescence (Fig. 3b, c and Supplementary Figure 1). These cells showed P2X_7_-dependent pore activity in response to 0.5 mM BzATP (Fig. 3d). Despite demonstrating increased surface expression of P2X_7_a, a decrease in BIL03s binding was observed (Fig. 3c), suggesting that high surface P2X_7_a expression may attenuate expression of nfP2X_7_ at the cell surface. These results show that BIL03s binds specifically to nfP2X_7_ and not to P2X_7_a, whereas L4 binding reflects the expression level of P2X_7_a but not nfP2X_7_.

To confirm that BIL03s does not bind to WT P2X_7_, we investigated the effect of two distinct siRNAs on BIL03s and L4 binding to PC3 or to PC3-P2X_7_a cells. Although siRNA A targets the coding sequence of *P2RX7* (Fig. 3e) and therefore targets both endogenous *P2RX7* and the *P2RX7* transgene, siRNA B targets the 3′-untranslated region and only downregulates endogenous *P2RX7*, as the *P2RX7* transgene lacks the 3′-untranslated region. As anticipated on the basis of the target sequence, siRNA A downregulated both L4 and BIL03s binding in PC3 and PC3-P2X_7_a cells. However, siRNA B downregulated BIL03s but not L4 binding, confirming that BIL03s recognises the product of the endogenous *P2RX7* gene and not the transfected *P2RX7* (Fig. 3f). Surprisingly, siRNA B knockdown caused an increase in L4 binding, although this was not statistically significant. E200 is adjacent to the ATP-binding pocket and could become exposed upon a change in receptor conformation caused by ATP binding. However, BIL03s and BPM09 binding were not affected by 1 mM ATP, demonstrating that ATP binding to P2X_7_ does not affect E200 exposure (Supplementary Figure 2). These data demonstrate that BIL03s and L4 bind to molecularly distinct forms of P2X_7_. Although L4 binds to the WT P2X_7_ associated with pore function, BIL03s binds to nfP2X_7_.

### nfP2X_7_ expression is induced by mimicking the high ATP concentration present in the TME

**Fig. 4:**
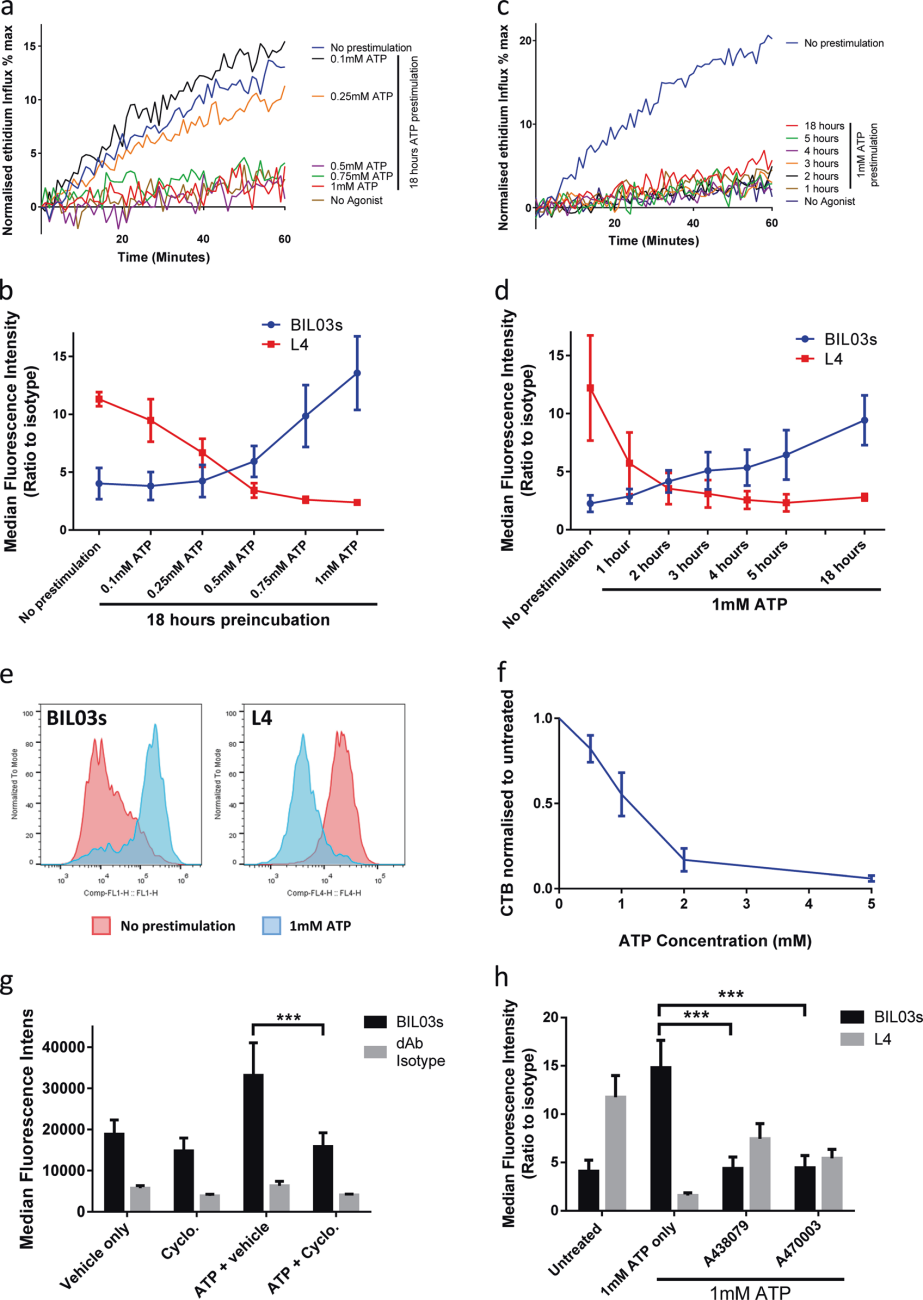
nfP2X7 expression can be induced by mimicking the high ATP concentration present in the tumour microenvironment. **a**, **b** RPMI-8226 cells were treated with ATP at the indicated concentration for 18 h. After treatment, **a** normalised ethidium influx in response to 0.5 mM BzATP stimulation was measured (mean of three independent experiments is shown) and **b** flow cytometry was used to quantify binding of BIL03s and L4 to live RPMI-8226 cells (mean and SEM from three independent experiments are shown). **c**, **d** RPMI-8226 cells were treated with 1 mM ATP for the indicated length of time. After treatment, **c** normalised ethidium influx in response to 0.5 mM BzATP stimulation was measured (mean from three independent experiments is shown) and **d** flow cytometry was used to quantify binding of BIL03s and L4 to live RPMI-8226 cells (mean and SEM from three independent experiments are shown). **e** Representative flow cytometry plots showing BIL03s and L4 binding to live RPMI-8226 cells before and after 1 mM ATP treatment for 18 h. **f** RPMI-8226 cells were treated with ATP at indicated concentration for 18 h before measurement of the number of live cells using CellTitre-Blue (CTB) assay. CTB fluorescence was normalised to untreated cells. Mean and SEM from three independent experiments are shown. **g** RPMI-8226 cells were treated with 250 μg/ml Cycloheximide, 1 mM ATP or both for 4 h. Flow cytometry analysis showed that increased BIL03s binding following 1 mM ATP treatment was abrogated in the presence of Cycloheximide. Mean and SEM from three independent experiments are shown. Two-way ANOVA with Dunnett’s post test was used to test significance. **h** RPMI-8226 cells were treated with 1 mM ATP alone or in the presence of P2X_7_ inhibitors A438079 or A740003 for 18 h. Flow cytometry analysis showed that the ATP-induced increase in BIL03s binding was significantly reduced by P2X_7_ inhibitors. Mean and SEM from three independent experiments are shown. Two-way ANOVA with Dunnett’s post test was used to test significance. ***P < 0.001

Studies have shown that nfP2X_7_ is highly expressed in tumour biopsies [25–27]. We hypothesised that high ATP concentrations present within the TME might drive this high expression. To test this hypothesis, we used RPMI-8226 cells to analyse the effect of overnight ATP incubation on BIL03s and L4 binding, and pore function (Fig. 4a, b). Overnight incubation with ATP in the 0.1–0.25 mM range led to a progressive decrease in pore function that was completely abrogated when the ATP concentration was raised to 0.5 mM (Fig. 4a). Decreased pore formation correlated with both a decrease in L4 binding and an increase of BIL03s binding (Fig. 4b). An increase in BIL03s binding was observed at ATP concentrations of 0.5 mM and above (Fig. 4b). These concentrations are similar to those measured in the TME [16]. Hence, RPMI-8226 cells adapt to high ATP concentrations by switching from P2X_7_ to pore-deficient nfP2X_7_. Representative population histograms show the change in staining for BIL03s and L4 between untreated cells and cells pre-stimulated with 1 mM ATP (Fig. 4e).

To assess the speed of change between P2X_7_ forms, we performed a time course in the presence of 1 mM ATP (Fig. 4c, d). One-hour stimulation with 1 mM ATP was sufficient to abrogate pore formation in response to BzATP agonist (Fig. 4c) and correlated with decreased L4 binding at 1 h, whereas BIL03s binding remained unchanged (Fig. 4d). L4 binding to RPMI-8226 cells decreased with time of ATP exposure with most binding abolished by 3 h. In contrast, BIL03s binding only increased 2 h after ATP incubation. Our data show that incubation with ATP promotes a rapid downregulation of WT P2X_7_, followed by a slower increase in surface nfP2X_7_.

nfP2X_7_ expression is transient and peaks at 24 h post ATP stimulation (Supplementary Figure. 3a). Induction of nfP2X_7_ in RPMI-8226 cells requires the presence of functional P2X_7_ at the cell surface as demonstrated by L4-binding data (Supplementary Figure. 3b). ATP treatment leads to a rapid decrease in WT P2X_7_ at the cell surface, which removes the drive for a further increase in nfP2X_7_. Other cellular stresses and danger-associated molecular patterns may also drive nfP2X_7_ exposure at the surface of cancer cells and are being investigated.

As opening of the P2X_7_ pore can be cytotoxic, it is possible that the ATP-mediated shift in L4 and BIL03s binding is the result of the selection of P2X_7_^low^/nfP2X_7_^high^ cell population. Overnight incubation of RPMI-8226 cells with 1 mM ATP led to 40%–50% loss of viability (Fig. 4f). However, the slower increase in nfP2X_7_ expression, measured by BIL03s binding, suggests that this could be protein synthesis dependent. To address this, we tested whether blocking protein synthesis would prevent increases in BIL03s binding. Figure 4g shows that cycloheximide treatment abrogated the ATP-induced increase in BIL03s binding at 4 h without affecting cell viability (data not shown), thus demonstrating that nfP2X_7_ induction is an active mechanism requiring protein synthesis and not mediated solely by selection of a P2X_7_^low^/nfP2X_7_^high^ sub-population.

RPMI-8226 cells were incubated in the presence of 1 mM ATP alone or with P2X_7_-selective inhibitors (A438079 or A740003) for 16 h, and BIL03s and L4 binding were measured. The ATP-induced increase in BIL03s binding and decrease in L4 binding were partially blocked by both P2X_7_ inhibitors, suggesting that the P2X_7_ to nfP2X_7_ switch required P2X_7_ signalling (Fig. 4h). Similar results were observed in the adherent melanoma cancer cell line SK-MEL-5 that also showed an ATP-induced increase in nfP2X_7_ expression, driven by a higher ATP concentration (5 mM) (Supplementary Figure 4).

### nfP2X_7_ is present intracellularly and localises to the membrane upon ATP stimulation

**Fig. 5:**
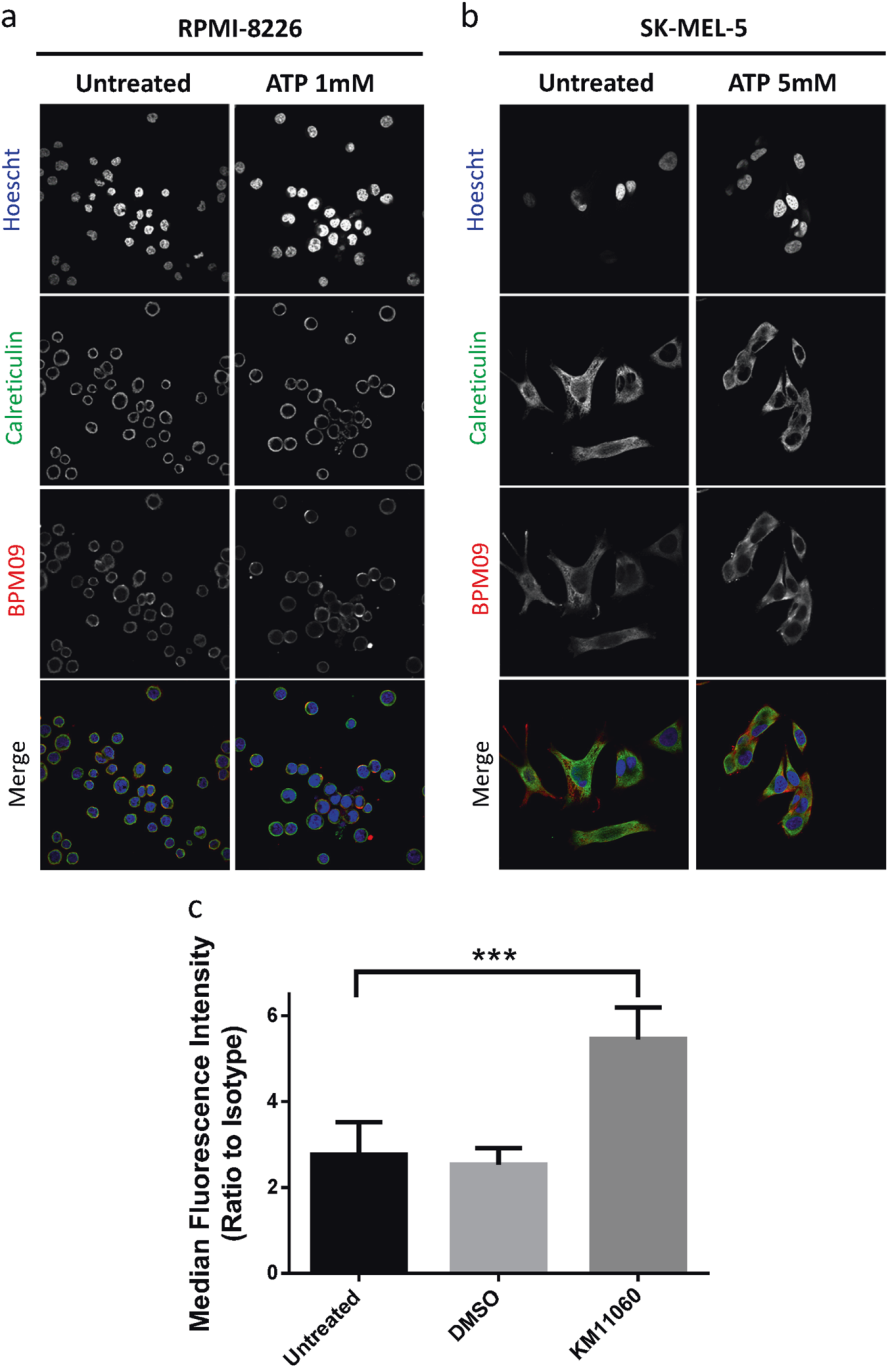
nfP2X7 is present intracellularly and addressed to the membrane upon ATP stimulation. **a**, **b** RPMI-8226 and SK-MEL-5 cells were treated with 1 mM ATP and 5 mM ATP, respectively, before performing immunofluorescence staining for BPM09 (7.6 μg/ml) and calreticulin (Abcam ab2907 2 μg/ml). Hoescht was used to stain the nucleus. Representative images from three independent experiments are shown. **c** RPMI-8226 cells were treated with 10 µM KM11060 or DMSO vehicle control for 16 h before analysing BIL03s and its isotype control binding by flow cytometry. Mean and SEM of three independent experiments are shown. One-way ANOVA with Dunnett’s post test was used to test significance. ***P < 0.001

To test whether nfP2X_7_ was present intracellularly, we investigated the cellular localisation of nfP2X_7_ by immunofluorescence. When comparing BPM09 detection with the known intracellular protein calreticulin, we found nfP2X_7_ to be present intracellularly and released to the membrane upon ATP stimulation (Fig. 5a, b). This suggests that nfP2X_7_ might correspond to a misfolded form of P2X_7_ normally retained intracellularly. A similar mechanism was described for mutated cystic fibrosis transmembrane receptor (CFTR) [33]. We tested this hypothesis using KM11060, a small molecule corrector, to increase the secretion rate of misfolded proteins to the membrane [33]. Treatment with KM11060 for a 16 h period led to nfP2X_7_ exposure at the cell surface (Fig. 5e). This suggests that nfP2X_7_ is sequestered intracellularly and could correspond to a misfolded form of P2X_7_.

### nfP2X_7_ is necessary for cancer cell survival

**Fig. 6:**
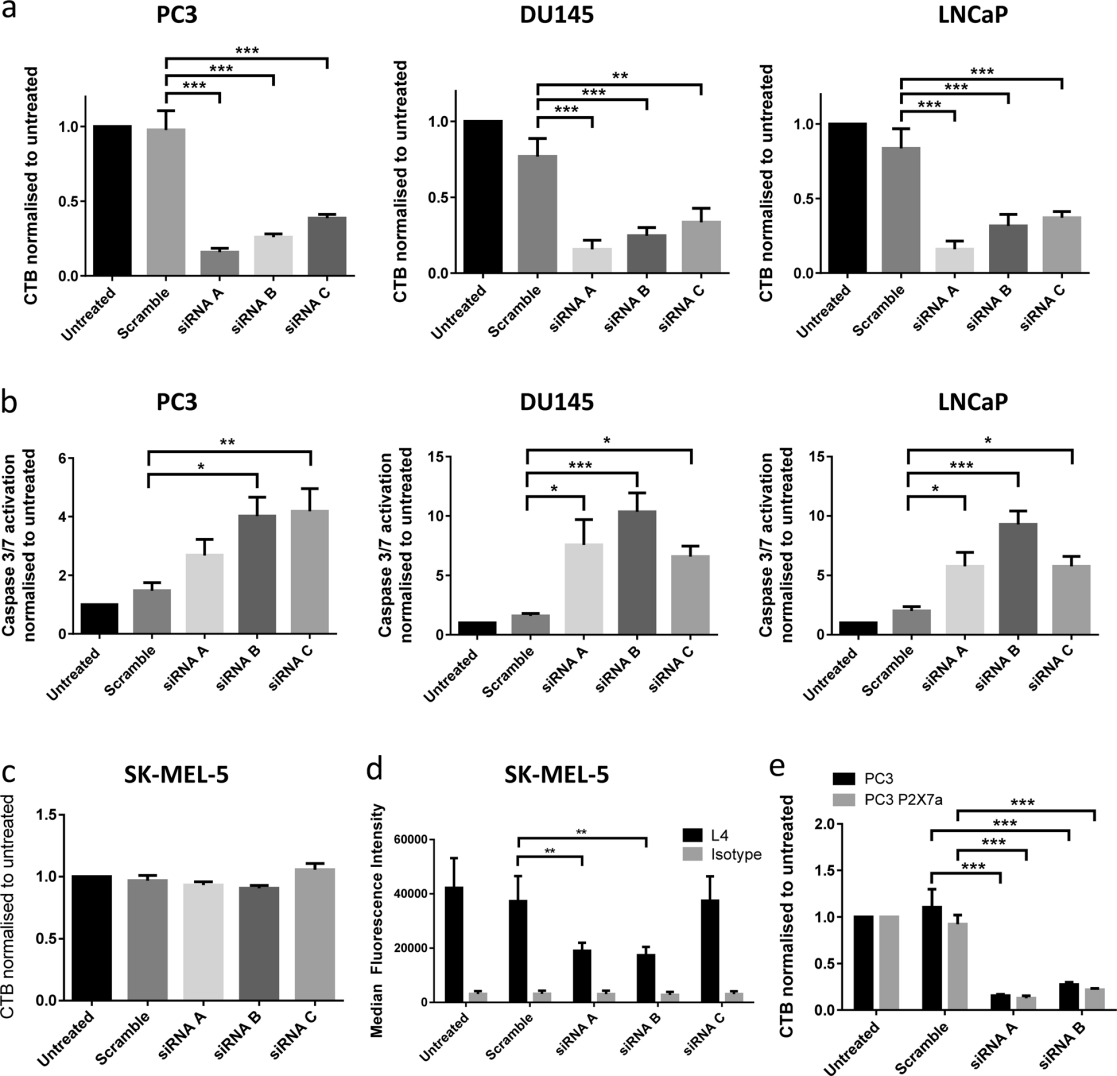
nfP2X_7_ is necessary for cancer cell survival. **a**, **b** PC3, DU145 and LNCaP non-functional cell lines were transfected with *P2RX7*-targeted siRNA. After 72 h, **a** number of live cells were measured using CellTitre-Blue (CTB) assay. **b** Induction of apoptosis was measured using an Apo One caspase 3/7 activation assay. Mean and SEM from at least four independent experiments shown. One-way ANOVA with Dunnett’s post test was used to test significance. **c**, **d** SK-MEL-5 functional cells were transfected with *P2RX7*-targeted siRNA. After 72 h, **c** no effect on cell viability was measured by CTB assay. **d** Surface expression of P2X_7_ as measured by L4 flow cytometry binding was significantly reduced. Mean and SEM from at least four independent experiments shown. Two-way ANOVA with Dunnett’s post test was used to test significance. **e** PC3 cells or PC3 cells overexpressing P2X_7_a were transfected with *P2RX7*-targeted siRNA. After 72 h, number of live cells was quantified using a CTB assay. *P2RX7*-targeted siRNA caused a reduction in cell numbers even in the presence of overexpressed P2X_7_a. Mean and SEM from at least four independent experiments shown. Two-way ANOVA with Dunnett’s post test was used to test significance. *P < 0.05, **P < 0.01, ***P < 0.001

To assess the role of nfP2X_7_ in cancer cell survival, we transfected three cell lines, devoid of P2X_7_ pore function and possessing nfP2X_7_ (PC3, DU145 and LNCaP), with three *P2RX7*-targeting siRNAs and measured the effect on cell viability. Treatment with each of the three *P2RX7*-targeted siRNAs caused significant cell death relative to control in all cell lines (Fig. 6a). We then showed that all *P2RX7*-targeted siRNAs tested caused a significant increase in Caspase 3/7 activation in each cell lines indicating that nfP2X_7_ depletion leads to apoptosis (Fig. 6b). These data demonstrate that in the absence of WT P2X_7_, nfP2X_7_ is essential for cancer cell survival.

We then investigated whether similar results could be obtained in cancer cell lines expressing high levels of WT P2X_7_. Although *P2RX7* siRNA significantly reduced L4 binding in SK-MEL-5, it did not have an effect on cell viability, suggesting that nfP2X_7_ but not WT P2X_7_ is required for cancer cell survival (Fig. 6c, d). We then transfected *P2RX7*-targeting siRNA A and B into both PC3 WT and PC3 overexpressing P2X_7_a (Fig. 6e). Loss of cell viability was not rescued when PC3 cells overexpressing P2X_7_a were transfected with siRNA B. Similar results were seen in the presence of increasing ATP concentrations (Supplementary Figure 5). These data demonstrate that nfP2X_7_ is necessary for cancer cell survival, and that loss of cell viability cannot be rescued by P2X_7_a. Therefore, although nfP2X_7_ has lost pore function, it retains a distinct pro-survival trophic activity.

### nfP2X_7_ is expressed in a broad range of tumours

**Fig. 7:**
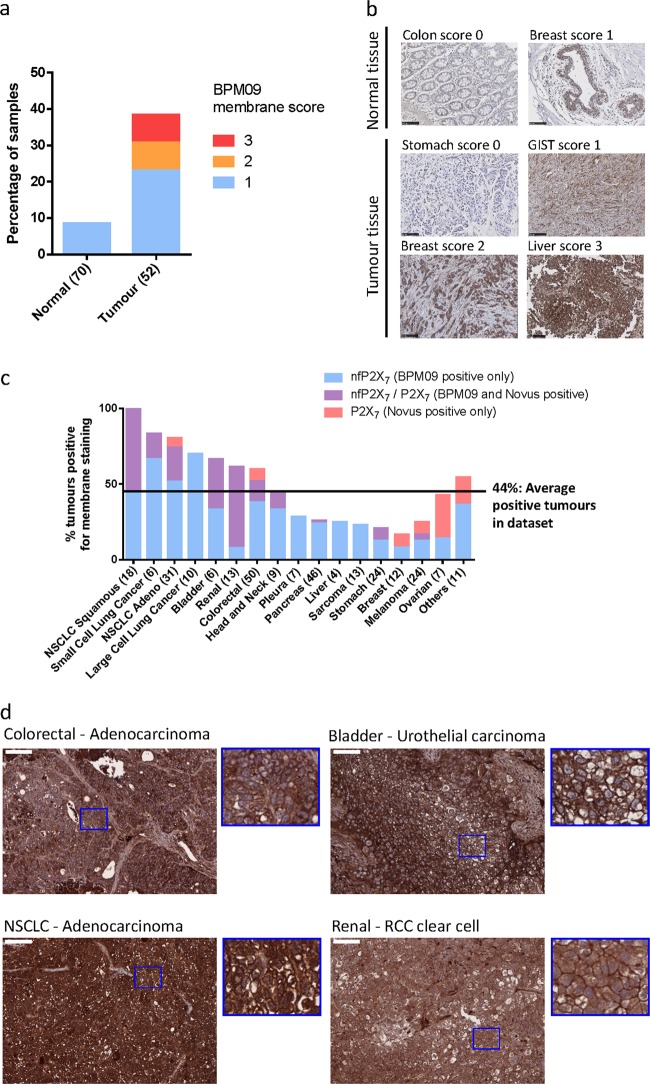
nfP2X_7_ is present in a broad range of tumour samples. **a** Tissue sections from 70 normal biopsies and 52 tumour biopsies were stained with BPM09 and scored for membrane staining. **b** Representative images for each score of normal and tumour tissue section stained with BPM09. Scale bar represents 100 µM. **c** Tissue sections from 290 Patient-derived xenograft (PDX) models were stained with BPM09 and a polyclonal antibody raised against part of the extracellular domain of P2X_7_a. Tissue sections were scored for membrane staining. For each tumour type the percentage of PDX with positive staining is shown with the number of PDX examined in brackets. **d** Representative images of colorectal (adenocarcinoma), bladder (urothelial carcinoma), non-small cell lung cancer (NSCLC—adenocarcinoma) and renal cancer (RCC clear cell) tissue sections with positive BPM09 staining together with higher magnification section showing BPM09 membrane staining. Scale bar represents 100 µm

To examine the potential of nfP2X_7_ as a therapeutic target, we assessed nfP2X_7_ membrane expression in tissue samples from a panel including 70 normal and 52 tumour samples. Weak membrane staining was observed in six normal tissues (< 10% of normal tissues), whereas 40% of tumour samples showed continuous positive staining (Fig. 7a, b). To assess nfP2X_7_ expression to normal cells, we performed flow cytometry analysis of peripheral blood mononuclear cell populations for BIL03s binding to monocytes, T- and B-cell lymphocytes. Data show weak BIL03s binding to the B-cell population and monocytes. However, BIL03s binding observed in leukocytes was much lower than observed in cancer cells (Supplementary Figure 6).

To address whether our in vitro observations were confirmed in vivo, we analysed P2X_7_ and nfP2X_7_ membrane expression in tissue samples from a panel of 290 patient-derived xenografts grown in immunocompromised mice. As L4 antibody is not suitable for immunohistochemical studies, we used a commercially sourced polyclonal antibody raised against part of the extracellular domain of P2X_7_a. P2X_7_ is detected in fewer samples than nfP2X_7_ (44% average positive tumours for nfP2X_7_ against 18% for P2X_7_) with higher nfP2X_7_ expression observed in bladder, kidney, colorectal and lung cancer including all of the 18 squamous non-small cell lung cancer (NSCLC) samples tested (Fig. 7c). With the exception of ovarian, all other tumour types show higher nfP2X_7_ expression. Varying overlap between P2X_7_ and nfP2X_7_ expression is seen between tumour types, whereas only nfP2X_7_ is detected in liver, large cell lung cancer, sarcoma and cancer of the pleura. Representative BPM09 staining shows examples of positive staining with clear membrane localisation at the surface of colorectal, bladder, NSCLC and renal cancer tissue sections (Fig. 7d). These data show that nfP2X_7_ is more broadly detected than P2X_7_ in tumour samples with varying overlap between P2X_7_ and nfP2X_7_ expression across tumour types.

## Discussion

**Fig. 8:**
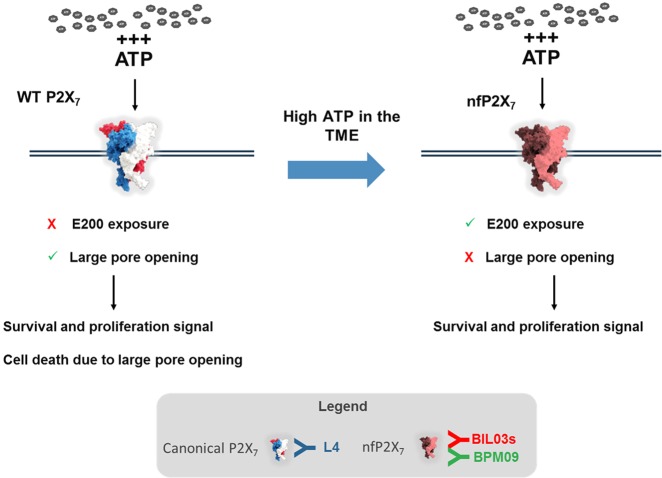
Illustration of the WT P2X_7_ to nfP2X_7_ switch and its impact on cell fate. L4 antibody binds specifically to WT P2X_7_ while BIL03s and BPM09 bind specifically to nfP2X_7_

In this study, we demonstrate for the first time that nfP2X_7_, characterised by exposure of E200 and loss of pore function, is distinct from functional P2X_7_ and is necessary for the survival of multiple cancer cell lines potentially through its retained ion channel function. We show that exposure to high ATP concentrations, similar to those present in the TME, facilitates a switch from P2X_7_ to nfP2X_7_ that enables cells to exploit the survival advantages of nfP2X_7_ signalling without the consequence of pore-mediated cell death (Fig. 8). Furthermore, our data confirms nfP2X_7_ is widely expressed at the surface of cancer cells and in a broad range of tumours.

P2X_7_ has been shown to support tumour growth and invasiveness in vivo in several cancer types [9, 34–36]. Previous reports have also shown that lack of or depletion of P2X_7_ causes or accelerates apoptosis in HEK293 and breast cancer cells [37, 38]. These studies neither investigate the functional status of P2X_7_ pore, nor the exposure of E200 on P2X_7_. Our data show that although nfP2X_7_ is unable to form a pore, it retains ion channel capability and supports cancer cell survival. Besides its trophic role, P2X_7_ promotes cytokine release [8, 39], activation of transcription factors [40] and adaption to serum-deprived conditions [12, 14]. It is possible that these functions contribute to nfP2X_7_-mediated cancer cell survival.

Converging evidence has shown that WT P2X_7_ can support cell proliferation [9, 14, 41]. This suggests that if cells are able to regulate opening of the P2X_7_ pore, they may exploit the trophic potential of P2X_7_, without the untoward effects of pore opening. Our data shows that WT P2X_7_ overexpressed in PC3 cells could not rescue cell death caused by knockdown of endogenous nfP2X_7_ even in the presence of agonist stimulation. This suggests that nfP2X_7_ provides a constitutively active survival signal, which cannot be replaced by WT P2X_7_. Although our results show that P2X_7_ and nfP2X_7_ are distinct, it is possible that nfP2X_7_ is present in heterotrimeric forms with WT P2X_7_, inhibiting its signalling as previously reported for P2X_7_ splice variant j [42]. Our data suggests that nfP2X_7_ is sequestered intracellularly and may correspond to a misfolded form of P2X_7_ as already reported for mutant forms of CFTR receptor (Fig. 5c). The observation that ATP-mediated E200 exposure requires protein synthesis (Fig. 4g) suggests that ATP signalling through P2X_7_ promotes machinery necessary for nfP2X_7_ localisation to the membrane. Furthermore, it is still debated whether opening of the large pore involves pannexin-1 or connexins. Our data show that WT P2X_7_ protein levels correlate with pore functionality but do not exclude a role for these proteins.

Several mechanisms are responsible for the elevated ATP concentration observed in the TME including dying tumour cells releasing ATP [16, 18]. This mechanism is supported by evidence from Ghiringhelli et al. [43] showing that breast cancer patients treated with adjuvant chemotherapy develop more aggressive metastatic disease if carrying the *P2RX7* mutation E496A, which abrogates pore function. Ghiringhelli et al. [43] showed that ATP released in the TME drives CD8^+^ T-cells priming by dendritic cells in a P2X_7_-dependent manner. Our data showing nfP2X_7_ expression in a subset of leucocytes suggest that nfP2X_7_ could also have a role in the regulation of immune cells in the TME. Although several mutations in the *P2RX7* gene have been reported to impair pore function [44], our data show that switching to E200 exposed nfP2X_7_ can provide a fast-adaptive mechanism to protect cancer cells from high ATP exposure.

A number of studies have demonstrated that nfP2X_7_ is expressed at the surface of several cancer types but is absent at the surface of normal tissue [23, 26, 27, 41, 45]. Using a panel of tissue specimens, we found nfP2X_7_ to be more widely detected than P2X_7_ at the membrane of tumour cells with positive immunohistological staining observed in approximately 40% of all samples tested. This study provides significant evidence that nfP2X_7_ is a distinct form of P2X_7_ with the potential to play a critical role in cell signalling leading to cancer cell survival. Further in vivo studies will be undertaken to establish further the role of nfP2X_7_ in cancer.

## Material and methods

### Reagent and antibodies

EtBr solution was purchased from Biorad. ATP, Fura-2 AM and phosphate-buffered saline (PBS)-based enzyme-free dissociation buffer were from Thermo Fisher Scientific. BzATP and A438079 were from Sigma. A740003 was from Tocris Bioscience. Monoclonal antibody clone L4 [31] was kindly provided by Professor James Wiley and Dr. Ben Gu.

### Cell culture

All cell lines were purchased from ATCC, except for LNCaP and Kelly, which were obtained from ECACC. All cell lines were authenticated by short tandem repeat analysis in October 2017. PC3 cells were cultured in F12K nutrient mixture Kaighn’s modification, 10% fetal calf serum (FCS). DU145 and SK-MEL-5 cells were cultured in minimum essential medium eagle, 10% FCS. LNCaP, Kelly and RPMI-8226 cells were cultured in RPMI medium 1640, 10% FCS.

#### Immunofluorescence

Cells were cultured on coverslips or on poly-lysine-coated coverslips for non-adherent cells, fixed with 4% paraformaldehyde in PBS, quenched with 0.1 M Glycine and permeabilised in 0.2% Saponin, 5% FCS in PBS. Cells were incubated with primary antibodies for 2 h at room temperature (RT) followed by secondary fluorescently labelled antibodies (Jackson Immunoresearch) for 1 h at RT. Confocal images were then acquired.

### Immunohistochemistry

Immunohistochemistry was performed on commercial Tissue Microarrays (TMAs) obtained from USBiomax, who obtained samples with full patient consent. BPM09 immunohistochemistry was performed as previously described [12, 27]. For anti-P2X_7_ (Novus: NBP2-19654), epitope retrieval was carried out in 10 mM Tri-Sodium Citrate/0.05% Tween 20, pH 6.0 for 20 min in a 600 W microwave before permeabilisation in 0.2% Triton X-100 for 15 min at RT and inactivation of endogenous peroxidase with a 3% hydrogen peroxide for 20 min at RT, blocking in 10% bovine serum albumin (BSA) at RT and staining overnight at 4 °C with 10 μg/ml of primary antibody. Each step was separated by tissue rinsing for 5 min in PBS. EnVision (Dako) was used as chromogen and haematoxylin counterstain. Slides were examined using a × 20 objective and scored by a pathologist for membranous staining.

### RNA interference transfection

A total of 200,000 cells were seeded in 6-well plates 16 h before transfection with 70 nM of relevant *P2RX7*-targeted siRNA from Origene using Lipofectamine RNAiMax. The siRNA target sequences were: siRNA A: 5′-CCCGCAGAGCAAAGGAATTCAGACC-3′, B: 5′-GAGATATTGTGAGGACAAATTGAGA-3′, C: 5′-ACAATGTTGAGAAACGGACTCTGAT-3′. AllStars scramble from Qiagen was used as a control. siRNA effect on cell survival was analysed using 2000 cells seeded in 96-well plates and transfected with 70 nM siRNA. 72 h after transfection, cell numbers were assessed using the CellTitre-Blue assay (Promega) or Apo One Caspase 3/7 assay (Promega).

### Real-time qPCR

Total RNA was extracted and cDNA prepared from 1 μg of RNA. qPCR was performed using primers for *P2RX7* (forward: 5′-TCTGCAAGATGTCAAGGGC-3′, reverse: 5′-TCACTCTTCGGAAACTCTTTCC-3′) and three housekeeping genes: β-actin (forward: 5′-CATCCTCACCCTGAAGTACC-3′, reverse: 5′-TTAATGTCACGCACGATTTC-3′), glyceraldehyde-3-phosphate dehydrogenase (forward: 5′-CTGCCGTCTAGAAAAACCTG-3′, reverse: 5′-GTCCAGGGGTCTTACTCCTT-3′) and β-2-microglobin (forward: 5′-ACTCACGTCATCCAGCAGAG-3′, reverse: 5′-TTCCCCCAAATTCTAAGCAG-3′). Triplicate qPCR reactions of 40 cycles were performed followed by melting curves. *P2RX7* transcript expression was normalised as previously reported [46]. Biological triplicates were tested for each sample.

### Ethidium influx assay

Fifty-thousand cells were seeded in 96-well plates 16 h before assay. Non-adherent cells RPMI-8226 and Ramos were seeded in poly-lysine-coated plates. Cells were washed twice with salt buffer (HEPES 10 mM, KCl 5.6 mM, Glucose 10 mM, NaCl 140 mM at pH 7.4) and incubated at 37 °C with 5% or 10% CO_2_ for 30 min. Cells were then treated with EtBr 25 μM and ATP or BzATP in salt buffer at 37 °C. Inhibitors were applied 5 min before the addition of agonist. Fluorescence (ex530–em620) was imaged every minute for 2 h at 37 °C on a plate reader. Cells permeabilised with 0.8% Triton were used for normalisation.

### Calcium assay

Changes in the cytoplasmic free calcium ([Ca^2+^]_i_) concentration were measured with fura-2/AM as described previously [47].

### ELISA assay

Streptavidin-coated 96-well plates were coated with 5 μg/ml of biotinylated E200 peptide for 16 h at 4 °C before 2 h blocking with PBS, 1% BSA and 1% Tween 20. Serial dilution of antibodies were incubated for 1 h at 4 °C before 1 h incubation with secondary antibody coupled to horseradish peroxidase and developing with tetramethylbenzidine reagent. Plates were washed three times in PBS 1% Tween 20 between each step.

### Flow cytometry

Fifty-thousand cells were dissociated in PBS-based enzyme-free dissociation buffer, washed and re-suspended in staining buffer (PBS, 2% FCS). Cells were then stained for 1 h with primary antibody, washed three times in staining buffer before a 1 h incubation with fluorescently coupled secondary antibody and 7-Amino Actinomycin D (7AAD). Fluorescence staining on live cells was acquired using a BD Accuri flow cytometer and analysed with FlowJo-flow cytometry analysis software. Median fluorescence intensity of the live cell population was analysed using 7AAD live dead staining.

### Western blotting

Cellular proteins were extracted using RIPA buffer supplemented with protease and phosphatase inhibitors cocktail from Roche Diagnostics. Equal protein amounts were analysed by SDS–polyacrylamide gel electrophoresis/western blotting using anti-P2X_7_ antibody from Alomone Labs (APR-004) and anti-actin antibody from Abcam (ab3280) as a loading control.

### Statistical analysis

Statistical analysis was performed using GraphPad. All figures show mean of at least three replicates unless otherwise indicated. Error bars represent SEM. Where significance is indicated, this has been calculated using analysis of variance with Dunnett’s multiple comparison test and Brown–Forsythe test to test for comparable variances unless otherwise indicated. *P < 0.05, **P < 0.01, ***P < 0.001.

## Electronic supplementary material

Supplementary Figure 1

Supplementary Figure 2

Supplementary Figure 3

Supplementary Figure 4

Supplementary Figure 5

Supplementary Figure 6

## References

[1] Burnstock G (2006). Purinergic signalling. Br J Pharmacol.

[2] North RA (2002). Molecular physiology of P2X receptors. Physiol Rev.

[3] North RA, Surprenant A (2000). Pharmacology of cloned P2X receptors. Annu Rev Pharmacol Toxicol.

[4] Surprenant A, Rassendren F, Kawashima E, North RA, Buell G (1996). The cytolytic P2Z receptor for extracellular ATP identified as a P2X receptor (P2X7). Science.

[5] Auger R, Motta I, Benihoud K, Ojcius DM, Kanellopoulos JM (2005). A role for mitogen-activated protein kinase(Erk1/2) activation and non-selective pore formation in P2X7 receptor-mediated thymocyte death. J Biol Chem.

[6] Buisman HP, Steinberg TH, Fischbarg J, Silverstein SC, Vogelzang SA, Ince C (1988). Extracellular ATP induces a large nonselective conductance in macrophage plasma membranes. Proc Natl Acad Sci USA.

[7] Di Virgilio F, Bronte V, Collavo D, Zanovello P (1989). Responses of mouse lymphocytes to extracellular adenosine 5’-triphosphate (ATP). Lymphocytes with cytotoxic activity are resistant to the permeabilizing effects of ATP. J Immunol.

[8] Adinolfi E, Callegari MG, Ferrari D, Bolognesi C, Minelli M, Wieckowski MR (2005). Basal activation of the P2X7 ATP receptor elevates mitochondrial calcium and potential, increases cellular ATP levels, and promotes serum-independent growth. Mol Biol Cell.

[9] Adinolfi E, Raffaghello L, Giuliani AL, Cavazzini L, Capece M, Chiozzi P (2012). Expression of P2X7 receptor increases in vivo tumor growth. Cancer Res.

[10] Di Virgilio F, Adinolfi E (2017). Extracellular purines, purinergic receptors and tumor growth. Oncogene.

[11] Jelassi B, Chantome A, Alcaraz-Perez F, Baroja-Mazo A, Cayuela ML, Pelegrin P (2011). P2X(7) receptor activation enhances SK3 channels- and cystein cathepsin-dependent cancer cells invasiveness. Oncogene.

[12] Adinolfi E, Melchiorri L, Falzoni S, Chiozzi P, Morelli A, Tieghi A (2002). P2X7 receptor expression in evolutive and indolent forms of chronic B lymphocytic leukemia. Blood.

[13] Amoroso F, Falzoni S, Adinolfi E, Ferrari D, Di Virgilio F (2012). The P2X7 receptor is a key modulator of aerobic glycolysis. Cell Death Dis.

[14] Baricordi OR, Melchiorri L, Adinolfi E, Falzoni S, Chiozzi P, Buell G (1999). Increased proliferation rate of lymphoid cells transfected with the P2X(7) ATP receptor. J Biol Chem.

[15] Allard B, Longhi MS, Robson SC, Stagg J (2017). The ectonucleotidases CD39 and CD73: novel checkpoint inhibitor targets. Immunol Rev.

[16] Pellegatti P, Raffaghello L, Bianchi G, Piccardi F, Pistoia V, Di Virgilio F (2008). Increased level of extracellular ATP at tumor sites: in vivo imaging with plasma membrane luciferase. PLoS ONE.

[17] Di Virgilio F, Dal Ben D, Sarti AC, Giuliani AL, Falzoni S (2017). The P2X7 receptor in infection and inflammation. Immunity.

[18] Kroemer G, Galluzzi L, Kepp O, Zitvogel L (2013). Immunogenic cell death in cancer therapy. Annu Rev Immunol.

[19] Lohman AW, Billaud M, Isakson BE (2012). Mechanisms of ATP release and signalling in the blood vessel wall. Cardiovasc Res.

[20] Raffaghello L, Chiozzi P, Falzoni S, Di Virgilio F, Pistoia V (2006). The P2X7 receptor sustains the growth of human neuroblastoma cells through a substance P-dependent mechanism. Cancer Res.

[21] Barden JA, Sluyter R, Gu BJ, Wiley JS (2003). Specific detection of non-functional human P2X(7) receptors in HEK293 cells and B-lymphocytes. FEBS Lett.

[22] Gu BJ, Zhang WY, Bendall LJ, Chessell IP, Buell GN, Wiley JS (2000). Expression of P2X(7) purinoceptors on human lymphocytes and monocytes: evidence for nonfunctional P2X(7) receptors. Am J Physiol Cell Physiol.

[23] Sluyter R. The P2X7 receptor. Adv Exp Med Biol 2017;1051:17–53. 10.1007/5584_2017_59 28676924

[24] Worthington RA, Smart ML, Gu BJ, Williams DA, Petrou S, Wiley JS (2002). Point mutations confer loss of ATP-induced human P2X(7) receptor function. FEBS Lett.

[25] Slater M, Scolyer RA, Gidley-Baird A, Thompson JF, Barden JA (2003). Increased expression of apoptotic markers in melanoma. Melanoma Res.

[26] Slater M, Danieletto S, Gidley-Baird A, Teh LC, Barden JA (2004). Early prostate cancer detected using expression of non-functional cytolytic P2X7 receptors. Histopathology.

[27] Slater M, Danieletto S, Pooley M, Cheng Teh L, Gidley-Baird A, Barden JA (2004). Differentiation between cancerous and normal hyperplastic lobules in breast lesions. Breast Cancer Res Treat.

[28] Gilbert SM, Gidley Baird A, Glazer S, Barden JA, Glazer A, Teh LC (2017). A phase I clinical trial demonstrates that nfP2X7 -targeted antibodies provide a novel, safe and tolerable topical therapy for basal cell carcinoma. Br J Dermatol.

[29] Donnelly-Roberts DL, Namovic MT, Han P, Jarvis MF (2009). Mammalian P2X7 receptor pharmacology: comparison of recombinant mouse, rat and human P2X7 receptors. Br J Pharmacol.

[30] Barden JA. Anti p2x7 receptor antibodies and fragments thereof WO2011020155 A1, 2011.

[31] Buell G, Chessell IP, Michel AD, Collo G, Salazzo M, Herren S (1998). Blockade of human P2X7 receptor function with a monoclonal antibody. Blood.

[32] Zhang XJ, Zheng GG, Ma XT, Yang YH, Li G, Rao Q (2004). Expression of P2X7 in human hematopoietic cell lines and leukemia patients. Leuk Res.

[33] Robert R, Carlile GW, Pavel C, Liu N, Anjos SM, Liao J (2008). Structural analog of sildenafil identified as a novel corrector of the F508del-CFTR trafficking defect. Mol Pharmacol.

[34] Amoroso F, Capece M, Rotondo A, Cangelosi D, Ferracin M, Franceschini A (2015). The P2X7 receptor is a key modulator of the PI3K/GSK3beta/VEGF signaling network: evidence in experimental neuroblastoma. Oncogene.

[35] Amoroso F, Salaro E, Falzoni S, Chiozzi P, Giuliani AL, Cavallesco G (2016). P2X7 targeting inhibits growth of human mesothelioma. Oncotarget.

[36] Qiu Y, Li WH, Zhang HQ, Liu Y, Tian XX, Fang WG (2014). P2X7 mediates ATP-driven invasiveness in prostate cancer cells. PLoS ONE.

[37] Adinolfi E, Callegari MG, Cirillo M, Pinton P, Giorgi C, Cavagna D (2009). Expression of the P2X7 receptor increases the Ca2+ content of the endoplasmic reticulum, activates NFATc1, andprotects from apoptosis.. J Biol Chem..

[38] Tan C, Han LI, Zou L, Luo C, Liu A, Sheng X (2015). Expression of P2X7R in breast cancer tissue and the induction of apoptosis by the gene-specific shRNA in MCF-7 cells. Exp Ther Med.

[39] Ferrari D, Chiozzi P, Falzoni S, Hanau S, Di Virgilio F (1997). Purinergic modulation of interleukin-1 beta release from microglial cells stimulated with bacterial endotoxin. J Exp Med.

[40] Ferrari D, Stroh C, Schulze-Osthoff K (1999). P2X7/P2Z purinoreceptor-mediated activation of transcription factor NFAT in microglial cells. J Biol Chem.

[41] Baricordi OR, Ferrari D, Melchiorri L, Chiozzi P, Hanau S, Chiari E (1996). An ATP-activated channel is involved in mitogenic stimulation of human T lymphocytes. Blood.

[42] Feng YH, Li X, Wang L, Zhou L, Gorodeski GI (2006). A truncated P2X7 receptor variant (P2X7-j) endogenously expressed in cervical cancer cells antagonizes the full-length P2X7 receptor through hetero-oligomerization. J Biol Chem.

[43] Ghiringhelli F, Apetoh L, Tesniere A, Aymeric L, Ma Y, Ortiz C (2009). Activation of the NLRP3 inflammasome in dendritic cells induces IL-1beta-dependent adaptive immunity against tumors. Nat Med.

[44] Wiley JS, Sluyter R, Gu BJ, Stokes L, Fuller SJ (2011). The human P2X7 receptor and its role in innate immunity. Tissue Antigens.

[45] Barden JA, Yuksel A, Pedersten J, Danieletto S, Delprado W (2014). Non-functional P2X7: a novel and ubiquitous target in human cancer. J Clin Cell Immunol.

[46] Vandesompele J, De Preter K, Pattyn F, Poppe B, Van Roy N, De Paepe (2002). Accurate normalization of real-time quantitative RT-PCR data by geometric averaging of multiple internal control genes. Genome Biol.

[47] Falzoni S, Munerati M, Ferrari D, Spisani S, Moretti S, Di Virgilio F (1995). The purinergic P2Z receptor of human macrophage cells. Characterization and possible physiological role. J Clin Invest.

