# Supplementary figures and images for: ATP in the tumour microenvironment drives expression of nfP2X7, a key mediator of cancer cell survival
Source: Oncogene. 2018 Aug 7;38(2):194–208. doi: 10.1038/s41388-018-0426-6 (PMC6328436; doi:10.1038/s41388-018-0426-6)

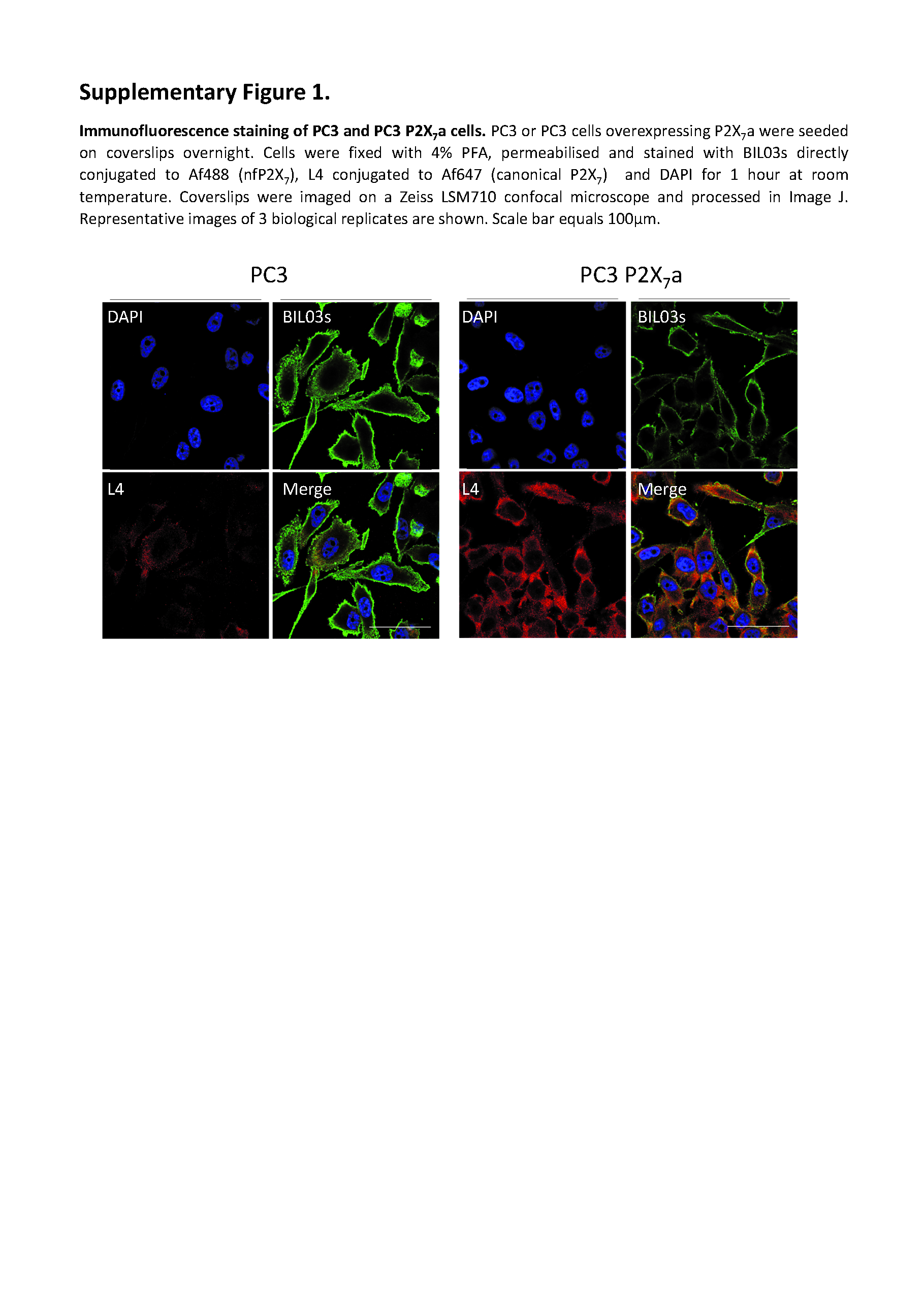

Supplement: Supplementary file 1 — Supplementary Figure 1 [file 41388_2018_426_MOESM1_ESM.tif]

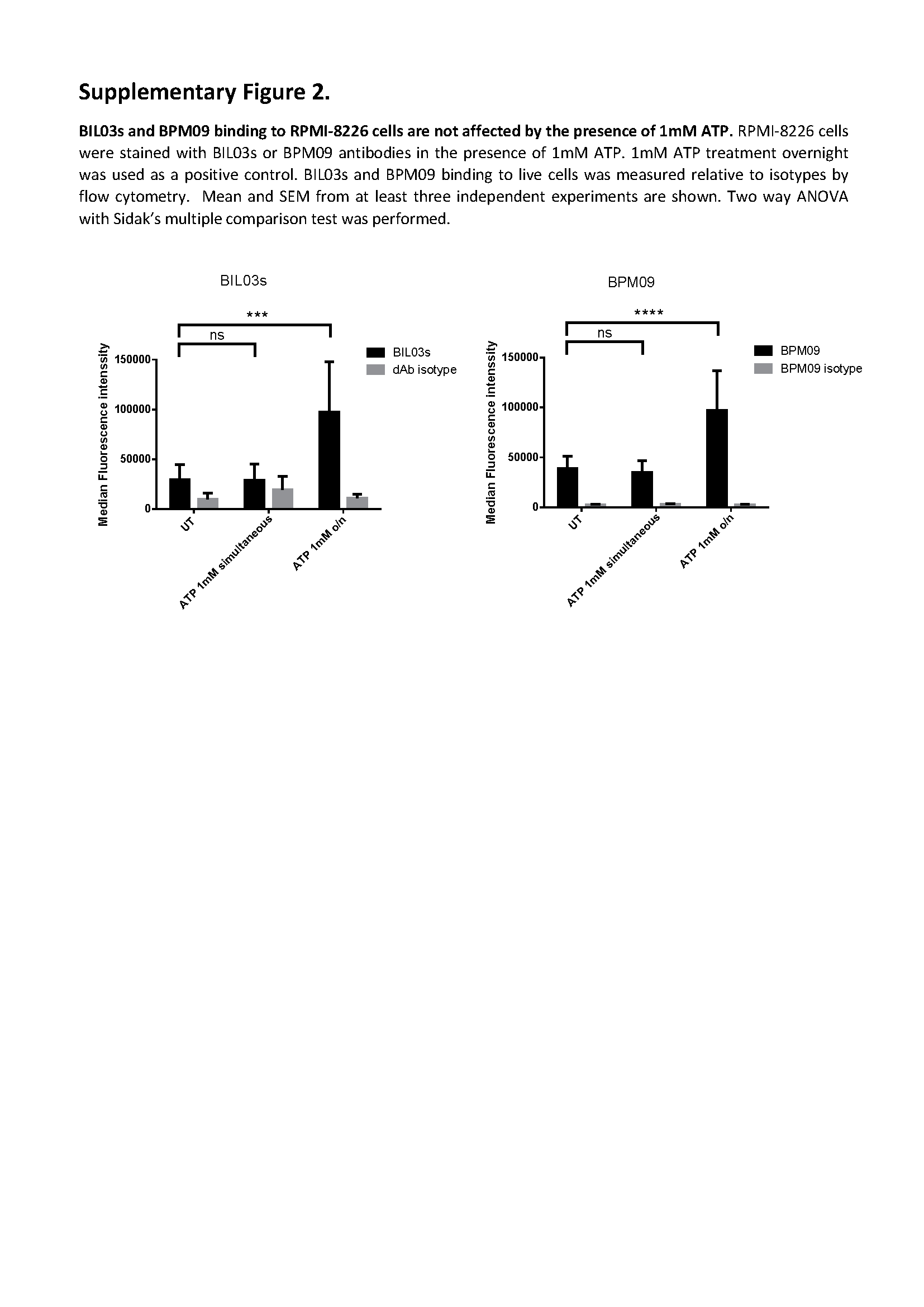

Supplement: Supplementary file 2 — Supplementary Figure 2 [file 41388_2018_426_MOESM2_ESM.tif]

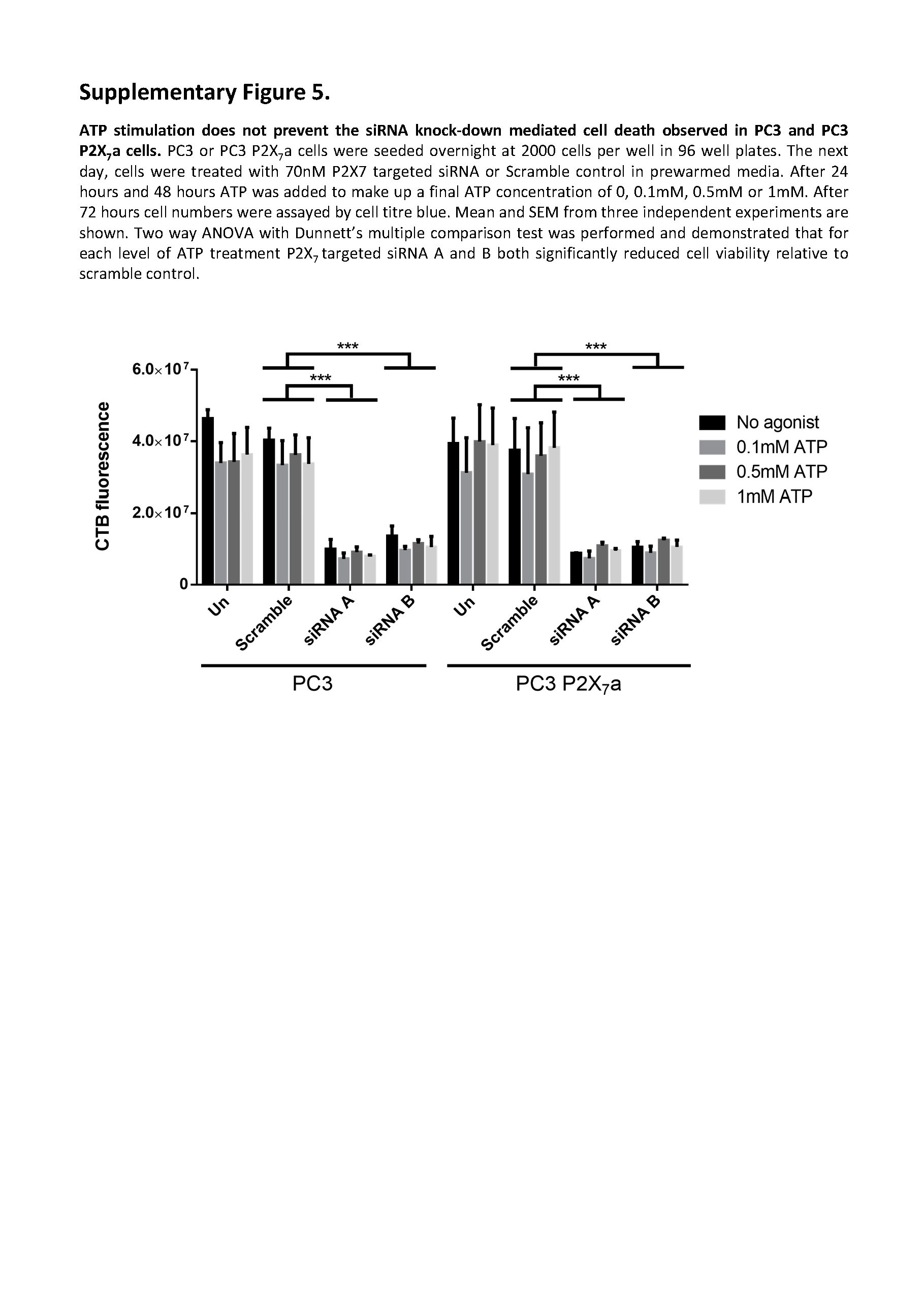

Supplement: Supplementary file 5 — Supplementary Figure 5 [file 41388_2018_426_MOESM5_ESM.tif]

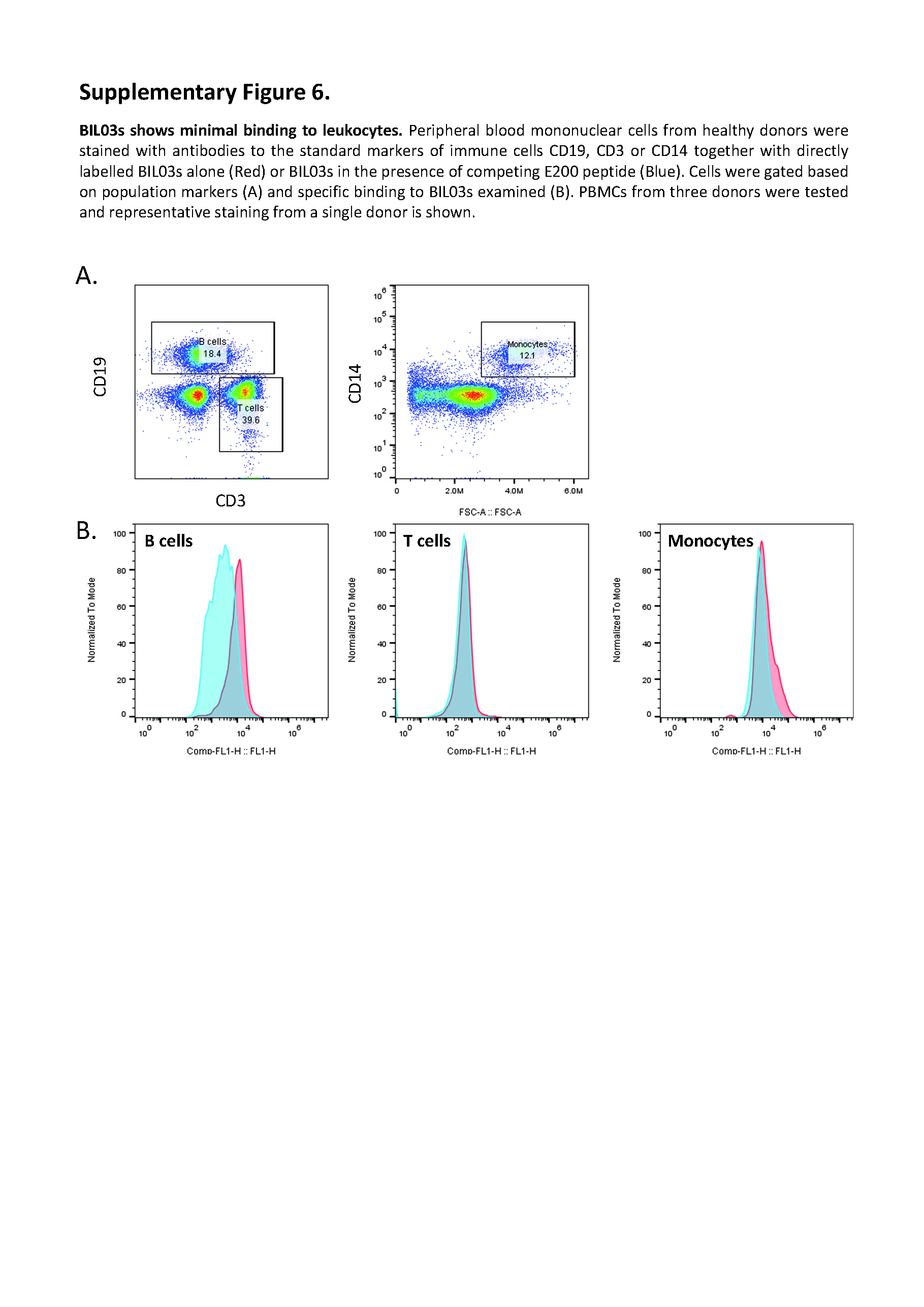

Supplement: Supplementary file 6 — Supplementary Figure 6 [file 41388_2018_426_MOESM6_ESM.tif]
